# Limited Stage Follicular Lymphoma: Current Role of Radiation Therapy

**DOI:** 10.4084/MJHID.2016.041

**Published:** 2016-09-01

**Authors:** Andrea Riccardo Filippi, Patrizia Ciammella, Umberto Ricardi

**Affiliations:** 1 Department of Oncology, University of Torino, Italy; 2 Department of Oncology and Advanced Technology, Arcispedale S Maria Nuova-IRCCS of Reggio Emilia, Italy

## Abstract

Radiation therapy (RT) alone has been considered for a long time as the standard therapeutic option for limited stage FL, due to its high efficacy in terms of local disease control with a quite significant proportion of “cured” patients (without further relapses at 10–15 years). Multiple therapeutic choices are currently accepted for the management of early stage FL at diagnosis, and better staging procedures as well as better systemic therapy partially modified the role of RT in this setting. RT has also changed in terms of prescribed dose as well as treatment volumes. In this review, we present and discuss the current role of RT for limited stage FL in light of the historical data and the modern RT concepts along with the possible combination with systemic therapy.

## Introduction

Approximately 25% of patients with follicular lymphoma (FL) present with stage I–II disease, the so-called “limited stage”, defined as either a single lymph nodal site or a limited lymphatic region without bone marrow involvement.^1^,^2^ For a long time, the preferred treatment approach has been radiation therapy (RT) alone, on the basis of several retrospective single-institution series showing a high rate of local disease control, with a proportion of patients (45%) achieving long-term survival without relapses (the only situation where FL has been defined as “curable”).^3^–^16^ However, there is a lack of prospective data, and very few retrospective studies have been conducted to compare this treatment modality versus others, including a “wait and see” policy. Recent advances in staging and new therapeutic options partially modified this scenario, and nowadays only 35–50% of patients are being offered RT alone at diagnosis in Unites States.^17^ This reflects a common pattern of practice among hematologists and radio-oncologists worldwide, given the extensive portfolio of therapeutic options. At the same time, RT has evolved towards the use of smaller fields and lower doses, with optimal control rates and minimal toxicity;^18^ data on the combination of low dose RT and rituximab became also available.^19^ Aim of this review is to present and discuss the current role of RT in this setting.

## History of RT Use and Current Indications

The definition of “limited” versus “generalized” FL depends on the definition of “limited” and by the intensity of staging investigations performed at diagnosis.^20^ Limited disease usually means stage I and contiguous stage II, as some stage II may be considered as generalized due to the presence of extended multiple sites disease (for example abdominal presentations). The presence of bone marrow involvement classically defines stage IV, however the presence of bone marrow minimal involvement (BCL-2/IgH rearrangement detected by polymerase chain reaction-PCR) has an uncertain significance. Approximately 60–80% of patients with presumed stage I–II disease may have circulating or bone marrow cells with Bcl-2/IgH rearrangement, with an unclear effect on prognosis.^21^ Most of the historical series reporting on outcomes following RT refer to a stage stratification based on clinical/radiological staging. An historical series by Goffinet et al. reported on 206 patients with “nodular” lymphoma where 31% of patients had stage I–II based on physical examination and imaging, but only 12% remained stage I–II after laparotomy/splenectomy for marrow negative patients.^22^ As the quality of imaging improved, together with the introduction of new modalities such as positron emission tomography (PET), a lower proportion of patients now present with stage II disease. In fact, truly localized disease is probably a rare entity, and few reports in the literature have sufficient magnitude for comparing clinical results after modern staging, since the patients’ accrual for most series took many years and the follow up interval for detecting relapses is at least 10 years.^20^ After RT the majority of the lesions completely regress, and local relapse at an irradiated site is rare. Recurrences usually occur distantly from the RT site and are rare after 10 years (1–11%). Probably the largest retrospective study on stage I or II FL included 568 patients diagnosed between 1973 and 2004, and was based on Surveillance Epidemiology and End Result (SEER) data.^23^ In 34 % of these patients, RT was used as the initial treatment; the group receiving RT at the onset had higher rates of disease-specific survival (DSS) at 5 years (90 vs. 81%), 10 years (79 vs. 66%), 15 years (68 vs. 57%), and 20 (63 vs. 51%) years, respectively. The rationale for the use of RT is thus based on the results of large mono-institutional experiences or observational cohort studies, which has been incorporated by international cooperative groups and clinical guidelines such as the National Comprehensive Cancer Network (NCCN), the European Society of Medical Oncology (ESMO) and the Italian Society of Hematology-Bone Marrow Transplantation Group (SIE-GITMO).^24^–^26^ Table 1 summarizes the results of the major studies on RT use for limited stage FL at diagnosis. Despite these indications, a recent observational study by the National Lymphocare project showed that variable treatment approaches are currently proposed to stage I–II FL patients: “wait and see” policy, chemotherapy, RT, Rituximab alone or systemic therapy plus RT.^17^ All these alternative treatments actually have excellent outcomes (median follow-up of 57 months). Interestingly, a subgroup of 206 patients who were rigorously staged, with bone marrow aspirate and biopsy and CT scan or PET scan or both, had increased survival. Among this patients’ cohort approximately 30% with confirmed stage I at PET-CT were offered RT alone as frontline therapy. With regards to the “wait and see” approach, researchers from Stanford University previously reported on a series of 43 selected patients with limited stage FL untreated at diagnosis who had a comparable outcome with those treated with RT alone.^27^ Soubeyran et al.^28^ also studied 43 patients with stage I follicular lymphoma been completely resected; 26 patients, accrued over an 11-years period, were selected for a “wait-and-see” policy (those had the lowest suspicion of residual disease): with a median follow up of 6.3 years, 13/26 (50%) relapsed, 6 locally and 7 distantly.

Nowadays, the evidence in favor of the use of RT at diagnosis for limited stage FL still relies on its curative potential: as shown in Table 1, historical series of more than 100 patients showed progression-free survival (PFS) rates at 10 years ranging from 40 to 59%, and overall survival rates of 58–86%.^5^,^7^,^9^,^10^,^14^,^15^ These series include patients treated as long ago as the 1970s, with implications regarding histological classification, staging procedure and systemic therapy. Despite the relative similarity of the outcome projections at 10 years, differences also exist among the reported series with regards to radiation volumes and dose, with a potential impact on late toxicity. The long-term survival outcome after RT alone for properly staged stage I–II FL patients, treated with modern fields and doses, is yet to be reported. Overall survival (OS) has apparently increased in recent years due to the introduction of Rituximab also for limited stage FL,^17^,^29^ but PFS after RT alone remained in this range, with approximately 45% of patients without relapses at 10 years.

## RT Volumes

From previously cited series, limited radiation volumes (involved field RT: IFRT) seem to be sufficient for disease control in limited stage FL. In fact, no differences in OS were reported by series including patients treated with either limited or more extensive radiation fields (e.g. extended fields RT (EFRT) or total nodal irradiation (TNI)). However, as expected, many retrospective studies observed that larger volumes resulted in higher PFS rates.^4^,^6^ For example, the Stanford University group showed that Total Lymphoid Irradiation (TLI) was associated with a lower relapse rate at 5 and 10 years (23% and 33%, respectively) compared with treatment to one side of the diaphragm only (52% and 64%, respectively).^4^ In these series, two thirds of deaths were due to other causes. At this regard, there are concerns that larger irradiation volumes might increase the risk of acute (e.g. hematological or gastrointestinal symptoms) and late (e.g. second tumors or cardiovascular disease) toxic effects.^9^ Wilder et al.^12^ found no differences in cause-specific survival (15 years: 59% vs. 72%) and OS (15 years: 49% vs. 40%) between patients treated with EFRT vs. IFRT. A large proportion of relapses (93%) involved the side of the diaphragm opposite the original site of disease and 59% exclusively affected the opposite side of the diaphragm. A German prospective multicenter phase II trial study investigated the influence of EF and total lymphoid irradiation on PFS, pattern of relapse and OS, showing no difference at 5 and 7 years between larger or smaller fields.^10^

IFRT for FL is traditionally defined as for Hodgkin’s lymphoma, including clearly defined “regions” of the Ann Arbor classification system,^18^ but a modern approach to more limited radiation fields has been developed in recent years following radical changes occurred for the radiation treatment of HL, introducing the so-called “involved nodal radiotherapy” (INRT) volumes.^30^ Unlike classic IFRT, INRT limits the treatment to only pre and post-chemotherapy involved nodal volumes. INRT is based on optimal pre-treatment imaging, taking into account pre-chemotherapy CT and FDG-PET scans. However, this concept does not simply apply to FL, as INRT was developed for Hodgkin’s Lymphoma (HL) for consolidation after chemotherapy, and not as a single modality curative approach. In FL, nodes that are “at risk” (i.e., minimally involved nodes that may be negative on PET imaging but involved by microscopic disease), should be included within radiation volumes in the absence of an active systemic therapy, as the likelihood of involvement is too great to be ignored.^20^ The International Lymphoma Radiation Oncology Group (ILROG) has thus developed specific consensus guidelines for the delineation of RT volumes for non-Hodgkin’s lymphomas that are slightly different from those developed for HL:^31^ the new concept, defined as “involved site radiotherapy” (ISRT), has been adopted by the NCCN and provides the basis for the current RT protocols.^24^ Although ISRT for NHL has not yet been validated through randomized trials, single-arm and retrospective data suggest comparable disease control to IFRT. A study from Vancouver looked retrospectively at patients that with limited stage FL treated either with IFRT or with a smaller field limited to the involved site.^32^ At a median follow-up of 7.5 years, both the larger and the smaller fields yielded excellent local control. Only 1% of patients who were treated with the smaller fields approach relapsed in adjacent regional nodes and there was no difference with respect to distant failure between the two groups (involved site 32 %, IFRT 38%).

In this context, the clinical target volumes identification for FL now requires consideration of quality/accuracy of imaging, expected patterns of spread, potential subclinical disease and adjacent organs at risk constraints. ISRT actually maintains the original intent of IFRT, but reduces the planned radiation volume trough an optimized use of modern imaging. This change in RT volumes reduces radiation exposure to organs at risk, reducing as well the risk of late toxicity.

## RT Dose

The curative radiation dose for localized FL has been in the range 36–45 Gy for a long time, as derived from early studies (Table 1). Within this dose range, local control reached approximately 90–95%, as already reported by Fuks and Kaplan in 1973.^33^ A series of studies from Princess Margaret Hospital further defined dose-response curves for both DLBCL and FL.^34^ For patients with medium or large-bulk disease (2.5–5 cm and >5 cm, respectively), 50% local control rate was achieved with a dose of 20 Gy, reaching 70% at 30 Gy and 80% at 40 Gy, with a plateau thereafter. For FL, doses in the range 25–35 Gy are able to obtain a local control rate >90%.^5^ Similar data were reported in a more contemporary series from University of Florida,^11^ with 30 Gy achieving local control in again >90% of patients. Stuschke et al.^10^ recommended a total dose of 30 Gy to lymph nodes with suspected subclinical disease and a total dose of 36–44 Gy to macroscopically involved lymph nodes. Nevertheless, in the “old” series many investigators noted that a significant number of patients with FL were controlled with a dose of <30 Gy.^35^ These findings led to the design of a randomized phase III study from the United Kingdom, comparing the standard dose of 40–45 Gy to 24 Gy in 361 involved sites of patients with indolent lymphomas (mostly FL and marginal zone B-cell lymphomas -MZL- in early stages).^36^ At a median follow-up of 5.6 years, there was no difference in overall response (93% and 92%, respectively) between the standard and the lower dose arms. There was also no difference in PFS or OS, and 24 Gy was thus established as the standard dose for treating limited-stage indolent non-Hodgkin’s lymphomas, including FL.

Very low-dose RT, largely used in the past years as palliation with total body irradiation, has also been proved to be effective in indolent NHL, particularly in FL. A radiation schedule of 4 Gy in 2 fractions was firstly shown to be highly effective when used for palliation of advanced-stage, relapsing, or even post multiple chemotherapy refractory patients with indolent lymphomas by Ganem et al.^37^ Girinsky et al. subsequently achieved an overall response rate (ORR) of 81%, with a median duration of response of 24 months and freedom from local progression of 56% at two years, on 48 patients classified as low-grade NHL according to the Working Formulation.^38^ A subsequent prospective study by Johanssonn et al. including 15 patients with FL confirmed a high ORR (87%), with a median duration of response of 22 months.^39^ A further study on 109 patients, including 98 FL, previously treated with multiple lines of chemotherapy, reported an ORR of 92%, and median duration of response and time to progression of 42 and 25 months, respectively.^40^ Murthy et al. also assessed the impact of low-dose RT on patients’ quality of life (European Organization for Research and Treatment of Cancer EORTC QLQ-C30), highlighting that low-dose RT was very well tolerated and had almost no impact.^41^ Regarding predictive/prognostic factors, Girinsky et al. reported lower PFS rates for patients previously treated with more than two chemotherapy lines, and lower response rates for masses larger than 5 cm and patients treated at age > 65 years.^38^ Haas et al.^40^ found no correlation between age, sex, follicular lymphoma grade, RT regimen, number of previous regimens and previous history, number of positive sites or largest lymphoma diameter and response rate; conversely, Russo et al.^42^ showed that patients aged <50 years had lower PFS rates (and also those with CLL histology in comparison with other indolent NHL subtypes).

Following these positive results, a randomized phase III trial was then conducted to compare standard dose (24 Gy/12 fractions) vs. low dose RT (4 Gy/2 fractions) as frontline radical or palliative treatment in FL and MZL.^43^ A total of 614 sites in 548 patients with FL (and some with MZL) were prospectively randomized to receive either 24 Gy or 4 Gy. In 60% of patients, the intent of RT was considered as palliative, and in 40% as curative. This study showed a higher ORR (81% vs. 74%) and 2-year PFS rate (94% vs. 80%) for 24 Gy vs. 4 Gy; thus 24 Gy remained the standard RT dose for the curative treatment of limited stage FL and MZL when radiation is administered as exclusive therapy. Table 2 summarizes the results of selected studies testing very low-dose RT for limited stage FL.

## Combination with Chemotherapy

As underlined in a recent comprehensive review,^20^ almost no studies reporting on the combination of RT and systemic therapy for stage I–II FCL were adequately powered to test for a difference in survival between RT vs. RT plus chemotherapy, given the rarity of early stage FCL. The British National Lymphoma Investigation (BNLI) conducted a trial between 1974 and 1980, where patients with Ann Arbor Stage I–II disease were treated with involved field RT alone to 35 Gy. Patients were then randomized to no further therapy (n=55) vs. chlorambucil 0.2 mg/kg/day orally for 8 weeks, followed by 0.1 mg/kg/day for16 weeks (n=50). No significant differences in PFS or OS were detected between the two arms.^44^ One large non-randomized trial has been reported by Seymour et al.,^45^ on 85 patients with stage I–II FL who received 3 cycles of chemotherapy followed by involved field RT (30–40 Gy) followed by cyclophosphamide, vincristine, prednisone, and bleomycin (COP-Bleo) for 7 cycles; patients with extra-nodal involvement, bulky disease (>5 cm.), or an elevated LDH also received doxorubicin (CHOP-Bleo). Ten-year freedom from treatment failure was 76%, and OS 82%. These appear both substantially better than results reported above for RT alone (however, this was not a randomized trial, and similar results have not been reported by others). Guadagnolo et al.^46^ reported on a series of 106 patients treated with IFRT +/− chemotherapy. There was no significant difference in PFS between patients who received chemotherapy and those who did not. The 10- and 15-year PFS rates were 47% and 43%, and 46% and 31% for patients treated with RT and with combined chemotherapy and RT, respectively (*p*=0.72). Patients were treated between 1972 and 2000, and interestingly, with very long-term follow-up, the incidence of secondary malignancies was not increased in this population in comparison with the expected incidence.

A retrospective series by Michallet et al. also reported a substantial equivalence in OS for patients treated at diagnosis with either chemotherapy-RT or RT alone; a possible explanation for the observed differences in PFS but not OS for the combination of chemotherapy and RT could be the good response rate at relapse to R-chemotherapy for patients who only received RT as first line therapy at diagnosis. OS was better for patients treated after the year 2000. A small group was also treated with chemoimmunotherapy upfront, with excellent results.^29^

## Combination with anti-CD20

As previously mentioned, the role of combined radio-chemotherapy in the management of limited-stage FL is uncertain, due to the reported significant toxicity, unclear superiority, and the fact that these studies were conducted in the pre-rituximab era. The introduction of the anti-CD20 antibody rituximab has radically changed the therapeutic options for patients with FL.^47^,^48^ Rituximab has been proposed as an alternative option to the watchful waiting approach in low-tumor burden advanced stage FL, and a recent multicenter randomized trial has shown the advantage of Rituximab vs. watch and wait for policy regarding PFS, although no advantages have been reported for OS.^49^ These promising results, as well as another phase II clinical trials, demonstrated a significant single agent activity of rituximab in both pretreated and untreated patients with FL.^50^

Furthermore, rituximab may contribute in eliminating the minimal residual disease in advanced disease^51^ and may enhance radiation-induced apoptosis and cell growth delay.^52^,^53^ The findings of these studies on advanced stage-low tumor burden FL cannot be directly extended to the limited stage. Nonetheless they provide the basis for a theoretically successful combination with RT for stage I–II disease, by increasing disease control outside radiation fields. At this regard, a case-cohort study by Ruella et al.^19^ showed for the first time that 4 doses of Rituximab followed by IFRT was a very well tolerated regimen able to reach a 10-year PFS rate of 64.6%, in comparison to the 50.7% rate achieved in control patients treated with RT alone for stage I–II FL (p<0.05). This superiority in PFS might translate into better long-term disease control and cure rate. Interestingly, this study also showed that among Rituximab-RT-treated patients, those with minimal bone marrow disease at baseline (PCR positivity) were at higher risk of relapse (6/10, 60%) compared to those with PCR negativity (4/23,17%), despite the use of Rituximab. This data stresses the importance of the evaluation and monitoring of molecular disease also in patients with low tumor burden, as this is probably one of the most important prognostic factors for relapse after both RT alone and RT-Rituximab. In fact, even with the addition of rituximab to RT, about 35% of patients do progress, and in particular patients with molecular disease positivity at diagnosis are at increased risk of relapse. Similar data were reported by Pulsoni et al. in a previous study, then updated.^54^,^55^ Fifty-seven consecutive patients treated with RT for limited stage FL were analyzed, and 38/57 (66.7%) had the molecular disease either in the bone marrow or peripheral blood despite a negative biopsy. Of these, 19/38 (50%) became negative after RT, and some patients with persistent positivity received Rituximab. However, the presence of molecular disease at diagnosis resulted to be associated with a worse prognosis despite the use of RT followed by Rituximab (10/11 relapses were PCR positive). Therefore, it could be reasonable in the future either to increase the initial dose of rituximab, with four additional doses after RT or to start maintenance at RT. This approach might be considered at least in patients presenting with the PCR-detectable disease at baseline. The MD Anderson Cancer Center is currently enrolling patients in a clinical trial offering a 2-yr maintenance after induction with rituximab-RT (NCT01473628). The MIR study, a phase II study of the German Low-Grade Lymphoma Study Group (GLSG), was designed with the first block of 4 rituximab doses, a 4 weeks gap with a restaging CT/planning CT of the involved nodal region in week 7 and then another block of 4 rituximab doses given concurrently with IFRT (40 Gy for macroscopic tumor or 30 Gy in case of CR). The primary endpoint of the study was PFS at two years, and preliminary data presented at the American Society of Hematology (ASH) 2012 meeting were encouraging, with a 2-yr PFS of 90%.^56^

## Conclusions and Future Perspectives

A moderate dose (24 Gy) Involved-site RT (ISRT) may cure approximately half of limited stage FL patients, with negligible acute and apparently no virtual late toxicity. Small radiation volumes are currently used for all localizations, including extra-nodal presentations. A high response rate is also achievable with very low dose RT (2 × 2 Gy), even in heavily pretreated patients; this option is now widely acknowledged to be very active as palliative treatment or frontline choice in selected cases. The combination of RT and anti-CD20 antibodies seems promising and might offer better long-term disease control; however, patients with molecular disease in the bone marrow or peripheral blood at diagnosis seem to be at high risk of relapse despite the use of RT plus rituximab. A trend towards a better survival is expected for patients staged with modern imaging, particularly with the use of CT-PET. Future perspectives in this field include the combination of RT with new targeted agents and immunotherapy, especially for those patients considered at higher risk of relapse; new generation anti-CD20 antibodies will probably further improve results. Limitations in the use of RT at diagnosis consist of the variety of therapeutic options for limited stage FL, including wait and see, without proven superiority of one modality vs. the other; given the rarity of the truly localized disease, it is unlikely that such data will become available over the next years. In consideration of its high efficacy (with a consistent proportion of patients without relapse at 15 years) and very low morbidity, modern RT maintains its role as a first choice treatment for the majority FL patients presenting with stage I–II disease at diagnosis.

## Figures and Tables

**Figure 1:**
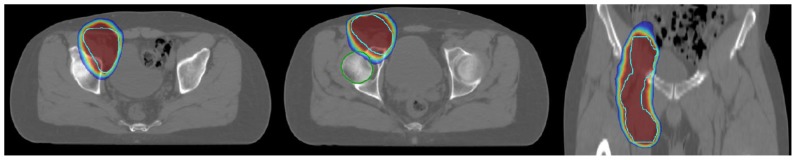
Example of involved site radiotherapy in a case of a 43 years old male patient affected with limited stage FL (right inguinal and crural nodes).

**Table 1:** Clinical outcomes of RT for limited stage FL.

Authors	Pts (n)	Total RT dose (Gy)	RT Volume	Follow-up yrs.	PFS (%)	OS (%)
Chen et al, 1979^3^	26	35–45	IF or EF		6-yr 83	NS
Paryani et al, 1983^4^	124	35–50	IF,EF,TNI	5,5	5-yr 62 10-yr 54 15-yr 42	5-yr 84 10-yr 68 15-yr 40
Gospodarowicz et al, 1984^5^	248	20–50 (<35 Gy in 86%)	IF	12	5-yr 56 10-yr 53	5-yr 73 10-yr 58
Epelbaum et al, 1992^6^	48	30–50	IF, EF	6,3	5-yr 71 10-yr 57	5-yr 83 10-yr 68
Vaughan Hudson et al, 1996^7^	208	35	NS	10	10-yr 47	10-yr CSS 71–84
Pendlebury et al, 1995^8^	58	30–54	IF,EF	NS	5-yr 59 10-yr 43	5-yr 93 10-yr 79
MacManus et al, 1996^9^	177	35–44	IF,EF,TNI	7,7	5-yr 55 10-yr 44 15-yr 40	5-yr 82 10-yr 64 15-yr 44
Stuschke et al, 1997^10^	117	26 + 10	EF,TNI	5,7	5-yr 71 10-yr 59	5-yr 86 10-yr 86
Kamath et al, 1999^11^	72	NS	IF,EF,TNI	NS	5-yr 62 10-yr 59 15-yr 47	5-yr 73 10-yr 46 15-yr 40
Wilder et al, 2001^12^	80	26–50	IF,EF	19	5-yr 63 10-yr 57 15-yr 41	5-yr 82 10-yr 65 15-yr 43
Ott et al, 2003^13^	58	26–50	IF,EF,TNI	8,8	5-yr 74 10-yr 64	5-yr 86 10-yr 69
Neumann et al, 2003^14^	116	20–50	IF,EF,TNI	4	5-yr 62 10-yr 48	5-yr 76 10-yr 51
Petersen et al, 2004^15^	460	16–47.5	IF	12.5	5-yr 56 10-yr 41	5-yr 79 10-yr 62
Eich et al, 2009^16^	65	26–46	IF,EF,TNI	9.1	5-yr 55 10-yr 37	5-yr 86 10-yr 55

Abbreviations: IF (involved field); EF (extended field); TNI (total nodal irradiation); NS (not specified); CSS (cancer specific survival).

**Table 2:** Studies on low dose RT for FL.

Authors	Pts (n)	Histology	Stage	Dose/fx	Response rate	Survival	Toxicity
Girinsky et al, 2001^38^	48	Low grade	I 15% II 23% III 27% IV 31%	4 Gy/2 fx	CR 57% PR 24%	Median duration of response: 24 months 2yr FFLP 56%	No events
Johannsson et al, 2002^39^	15	Indolent NHL	Advanced	4 Gy/2 fx	CR 74% PR 13%	Median duration of response: 22 months	No events
Haas et al, 2003^40^	109	Indolent NHL (FL=98),	Advanced (52%bulky)	4 Gy/2 fx	CR 61% PR 31% SD 6% PD 2%	Median duration of response: 42 months Median TTLP: 25 months PFS1yr 50% PFS2yrs 33% PFS3yrs 25% PFS5yrs 10%	No events
Murthy et al, 2008^41^	29	Indolent NHL	Advanced	4 Gy/2 fx	ORR 86%	NR	No events > G2
Russo et al, 2012^42^	127	Indolent NHL (including CLL)	I (16%) II (10%) III (31%) IV 43%)	4 Gy/2 fx	CR 57% PR 25%	TTP 13.6 months	No events

Abbreviations: NHL (non-Hodgkin’s lymphoma); FFLP (freedom from local progression); TTLP (time to local progression); PFS (progression free survival); CLL (chronic lymphatic leukemia); CR (complete response); PR (partial response); SD (stable disease); PD (progressive disease), ORR (overall response rate); NR (not reported).
